# New Books

**Published:** 2009-05

**Arsenic Pollution: A Global Synthesis**

Peter Ravenscroft, Hugh Brammer, Keith Richards

Hoboken, NJ: John Wiley & Sons, 2009. 616 pp. ISBN: 978-1-4051-8602-5, $129.95

**Diagnosis: Mercury: Money, Politics, and Poison**

Jane Hightower

Washington, DC: Island Press, 2008. 326 pp. ISBN: 978-1-59726-395-5, $24.95

**Doing Science: Design, Analysis, and Communication of Scientific Research**

Ivan Valiela

New York: Oxford University Press, 2009. 304 pp. ISBN: 978-0-19-538573-1, $34.95

**Globalization: The Making of World Society**

Frank J. Lechner

Hoboken, NJ: John Wiley & Sons, 2009. 336 pp. ISBN: 978-1-4051-6906-6, $89.95

**Green Urbanism Down Under: Learning from Sustainable Communities in Australia**

Timothy Beatley, Peter Newman

Washington, DC: Island Press, 2008. 280 pp. ISBN: 978-1-59726-411-2, $70

**Informing Decisions in a Changing Climate**

Panel on Strategies and Methods for Climate-Related Decision Support; National Research Council

Washington, DC: National Academies Press, 2009. 200 pp. ISBN: 978-0-309-13748-5, $39

**Mapping the Future of Biology: Evolving Concepts and Theories**

Anouk Barberousse, Michel Morange, Thomas Pradeu, eds.

New York: Springer, 2009. 184 pp. ISBN: 978-1-4020-9635-8, $129

**Microarrays: Preparation, Microfluidics, Detection Methods, and Biological Applications**

Kilian Dill, Robin Hui Liu, Piotr Grodzinsky, eds.

New York: Springer, 2009. 356 pp. ISBN: 978-0-387-72716-5, $149

**Principles of Ecosystem Stewardship: Resilience-Based Natural Resource Management in a Changing World**

F. Stuart Chapin III, Gary P. Kofinas, Carl Folke, eds.

New York: Springer, 2009. 402 pp. ISBN: 978-0-387-73032-5, $49.95

**Real World Ecology**

ShiLi Miao, Susan Carstenn, Martha Nungesser, eds.

New York: Springer, 2009. 312 pp. ISBN: 978-0-387-77916-4, $49.95

**Resilient Cities: Responding to Peak Oil and Climate Change**

Peter Newman, Timothy Beatley, Heather Boyer

Washington, DC: Island Press, 2009. 166 pp. ISBN: 978-1-59726-498-3, $60

**Restructuring Federal Climate Research to Meet the Challenges of Climate Change**

Committee on Strategic Advice on the U.S. Climate Change Science Program; National Research Council

Washington, DC: National Academies Press, 2009. 266 pp. ISBN: 978-0-309-13173-5, $50.18

**The Access Principle: The Case for Open Access to Research and Scholarship**

John Willinsky

Cambridge, MA: MIT Press, 2009. 307 pp. ISBN: 978-0-262-51266-4, $16.95

